# The Association Between Linguistic Characteristics of Physicians’ Communication and Their Economic Returns: Mixed Method Study

**DOI:** 10.2196/42850

**Published:** 2024-01-11

**Authors:** Shuang Geng, Yuqin He, Liezhen Duan, Chen Yang, Xusheng Wu, Gemin Liang, Ben Niu

**Affiliations:** 1 College of Management Shenzhen University Shenzhen China; 2 Shenzhen Health Development Research and Data Management Center Shenzhen China

**Keywords:** web-based health care, instrumental communication, affective communication, linguistic features, economic returns, linguistic inquiry and word count

## Abstract

**Background:**

Web-based health care has the potential to improve health care access and convenience for patients with limited mobility, but its success depends on active physician participation. The economic returns of internet-based health care initiatives are an important factor that can motivate physicians to continue their participation. Although several studies have examined the communication patterns and influences of web-based health consultations, the correlation between physicians’ communication characteristics and their economic returns remains unexplored.

**Objective:**

This study aims to investigate how the linguistic features of 2 modes of physician-patient communication, instrumental and affective, determine the physician’s economic returns, measured by the honorarium their patients agree to pay per consultation. We also examined the moderating effects of communication media (web-based text messages and voice messages) and the compounding effects of different communication features on economic returns.

**Methods:**

We collected 40,563 web-based consultations from 528 physicians across 4 disease specialties on a large, web-based health care platform in China. Communication features were extracted using linguistic inquiry and word count, and we used multivariable linear regression and K-means clustering to analyze the data.

**Results:**

We found that the use of cognitive processing language (ie, words related to insight, causation, tentativeness, and certainty) in instrumental communication and positive emotion–related words in affective communication were positively associated with the economic returns of physicians. However, the extensive use of discrepancy-related words could generate adverse effects. We also found that the use of voice messages for service delivery magnified the effects of cognitive processing language but did not moderate the effects of affective processing language. The highest economic returns were associated with consultations in which the physicians used few expressions related to negative emotion; used more terms associated with positive emotions; and later, used instrumental communication language.

**Conclusions:**

Our study provides empirical evidence about the relationship between physicians’ communication characteristics and their economic returns. It contributes to a better understanding of patient-physician interactions from a professional-client perspective and has practical implications for physicians and web-based health care platform executives.

## Introduction

### Background

Web-based health care platforms offer environments where patients can consult physicians and pay for their services remotely. These platforms are particularly helpful for patients residing in rural areas with limited access to medical resources and patients with limited mobility [1]. Physicians also benefit from providing web-based consultations, both in terms of economic returns and social returns, such as improving their reputation [2]. In recent years, web-based health care has been upheld as a national health priority in China. The number of web-based hospitals surged to 2700, and the number of users surged to 360 million in 2022 [3]. Physicians affiliated with offline hospitals can also provide services on third-party health care platforms, such as the Dingxiang Doctor and Haodf websites [4,5]. Unlike offline service prices, web-based consultation prices are not governed by certain pricing standards on these third-party health care platforms [6]. Thus, physicians can charge higher or lower fees per web-based consultation than their offline consultations. Meanwhile, patients can register as platform users, consult physicians, and make payment. Most of these health care platforms offer both synchronous telemedicine and asynchronous, message-based consultation services. We focus specifically on asynchronous, message-based consultations on the third-party health care platforms.

The quality of patient-physician interactions is vital for consultation outcome and patient satisfaction [7,8]. Although many studies have investigated the communication styles and features during web-based health care consultation [7-10], scant attention has been devoted to its associations with the economic returns of physicians. Characterizing physician-patient communications at a more granular level and exploring their associations with physicians’ economic returns can provide important guidance for physicians to improve their communication skills and economic benefits. The findings also have implications for the amelioration of platform incentive mechanisms.

Previous studies have identified 2 types of physician-patient interactions during web-based consultations: affective, which focuses on expressing care for the patients [8], and instrumental, which focuses on addressing the patients’ health problems [11]. Instrumental interactions typically involve discussions about the severity of the disease or syndrome, its causes, and potential treatment plans [12,13]. They are problem-solving oriented and emphasize the provision of medical information and expertise to patients. These 2 types of interactions are the 2 components of the professional-client interaction theory proposed by Ben-Sira [11] and are crucial for effective web-based health care consultations [14].

This study investigated the associations between these 2 types of interactions and the economic returns of physicians in the context of the Chinese web-based health care system. Specifically, we focused on asynchronous, text message–based consultation services, in which physicians are paid on a per-consultation basis. Physicians usually receive a larger portion (ie, 90%) of the consultation fee, with the remaining portion paid to the platform. We measured the economic returns of the physician as the consultation payment on a per-consultation basis. On the one hand, the consultation prices are initially established by physicians and range from RMB 30 (US $4.19) to RMB 699 (US $97.65) [4]. However, patients can make a choice among physicians, and they are only willing to pay a higher price for better service. Thus, physicians need to adjust the consultation prices to maintain the patient base, and the economic returns are regarded as being mutually determined by the patient and physician. During the consultation process, physicians can choose to use text or voice message for service delivery. Therefore, besides the association between communication characteristics and economic returns, we were also interested in how the associations change vis-à-vis different communication media (text vs voice).

### Instrumental and Affective Linguistic Features

Communication quality is critical for patient satisfaction and effective use of medical resources [15]. Previous studies have investigated the quality of patient-physician interactions using self-reported survey [7,16-18], statistical method [9,19,20], and manual labeling combined with text classification [14]. We derived insights from sociopsychological studies and used Linguistic Inquiry and Word Count (LIWC) to extract the linguistic indicators. LIWC provides a psychometrically validated dictionary comprising approximately 6400 words, word stems, and selected emoticons [21,22]. So far, LIWC has been widely used to identify emotional and cognitive dimensions in social, health care, and personality psychology research [23-26]. For instance, Liu et al [27] used LIWC to ascertain positive emotion words in replies provided by physicians to indicate their emotional support to patients.

**Figure 1:**
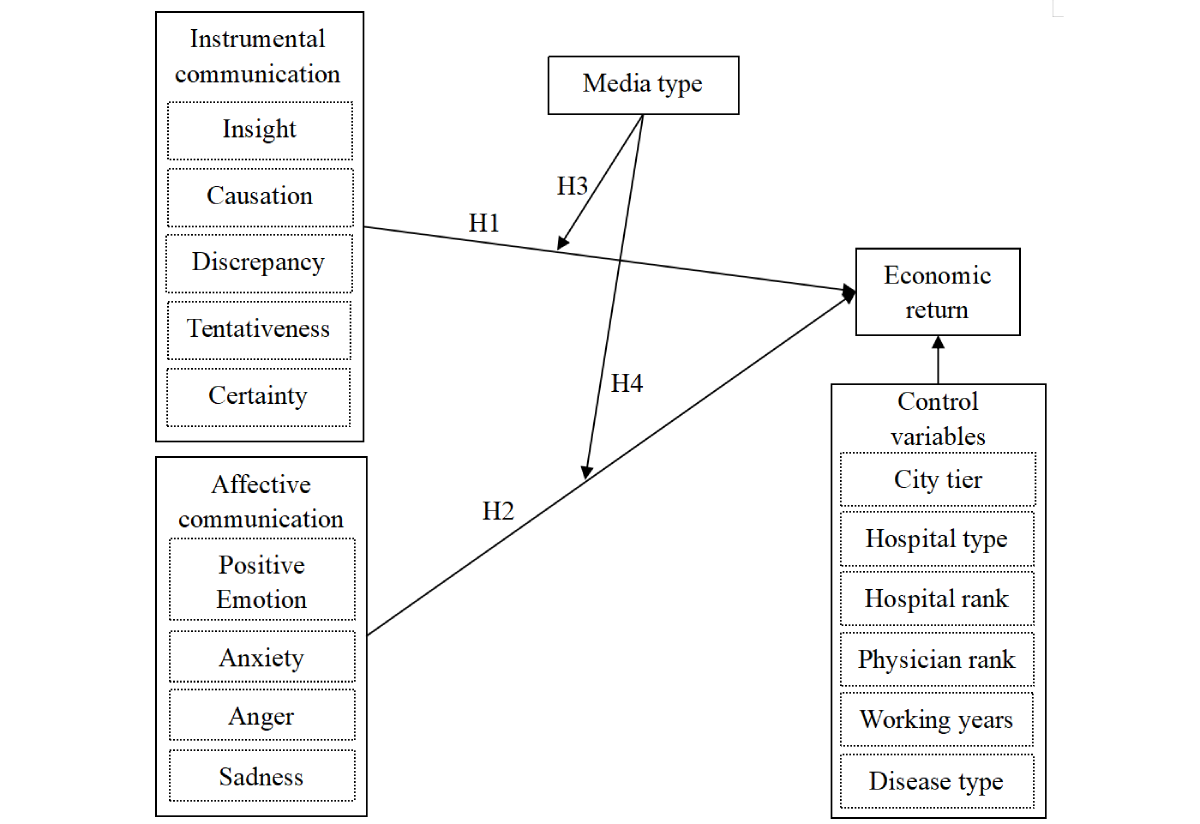
The research model. H1: hypothesis 1; H2: hypothesis 2; H3: hypothesis 3; H4: hypothesis 4.

In instrumental interactions, physicians demonstrate their expertise and cognitive thinking process. Hence, physicians tend to use words related to the cognitive process, presenting linguistic features regarding insight, causation, discrepancy, tentativeness, and certainty to deliver disease knowledge to patients [28]. Affective interactions articulate the emotions of physicians including the emotional support they provide to their patients to alleviate patient anxiety [29]. Notably, the emotional expressions of physicians could comprise diverse feelings such as happiness, anxiety, anger, and sadness. Thus, we included both positive and negative emotional linguistic features for affective interactions. Specifically, we use a simplified Chinese LIWC dictionary established according to the LIWC lexicon and its Chinese version [30-32]. In total, there are 9 linguistic features for the 2 types of interactions, as shown in Figure 1. Example words for each feature are provided in Multimedia Appendix 1.

## Methods

### Research Model

Figure 1 displays our conceptual model. This study explored the associations between the communication behaviors of physicians and their economic returns in 3 stages. First, it probed the impact of instrumental and affective communication of physicians on their economic returns. Second, it investigated the potential moderating effects of communication media (voice vs text). Third, it investigated the interaction patterns among different communication features and identified the pattern yielding the highest economic returns.

### Instrumental Interactions by Physicians and Their Economic Returns

Patients seek knowledge related to their health conditions through consultations with physicians because of their professional capital [33]. Thus, physicians are expected to provide disease-specific information, suggestions, or guidance to patients to improve communication quality and patient satisfaction. Physicians’ instrumental behaviors manifest mainly through the use of words related to the cognitive process that facilitate the inculcation of due medical knowledge in patients [34]. Thus, instrumental interactions are vital for physicians to achieve a consensus with their patients regarding relevant disease knowledge, treatment plans, and problem solutions.

Economic returns incentivize physicians by satisfying their financial needs. In our research context, health care platforms allow physicians to levy consultation charges as they deem fit [6]; patients can also offer monetary gifts to their physicians. According to the capital exchange theory [2], physicians use their decision capital to perform exchange actions. To a certain degree, the consultation service charges reflect the self-evaluation of physicians vis-à-vis their decision capital and service quality. In contrast, patients may attribute high expectations for the consultation process and outcomes when they agree to pay high service charges. Therefore, we postulated that high economic returns could correlate to more intense instrumental interactions. Specifically, we hypothesized the following: The instrumental communication features of physicians positively influence their economic returns (hypothesis 1).

### Affective Interactions by Physicians and Their Economic Returns

Physicians’ affective communications involve the expression of care about the feelings of their patients as human beings rather than medical cases [11]. During the consultation process, patients are often emotionally occupied and could unintentionally express their emotional concerns. Many studies have emphasized the psychological needs of patients, citing anxiety and distress as critical issues for health care services [7,8,14,35]. The expression of feelings such as empathy, sympathy, caring, and concern to patients could help mitigate the stress or anxiety induced in patients by their health issues [36]. Hence, patient satisfaction is also associated with the affective behaviors of physicians. In addition, many patients are ignorant of the complexities and technical challenges of their treatment solutions [6,11]. Hence, affective behaviors by physicians can influence patients’ perceptions about their service quality. Patients may place higher expectations for web-based consultation with higher charges in terms of the sensed emotional support. Therefore, we hypothesized the following: The affective communication features of physicians positively influence their economic returns (hypothesis 2).

### The Moderating Effects of Communication Media

Physicians and patients communicate primarily via textual and vocal means in asynchronous web-based health consultations. The media synchronicity theory asserts that media characteristics function significantly in information transmission and processing [37]. Vocal communication can deliver the speech signals of physicians, such as pronunciation and intonation, which textual communication using character data cannot [38]. Therefore, vocal communication allows patients to discern the subtleties of the thoughts or concerns of their physicians about their diseases. Moreover, vocal communication also adds to the multiplicity of cues to support complex tasks [14]. In addition, physicians can use vocal communication to deliver immediate and convenient feedback because typing messages is more time consuming. However, it cannot be presumed that vocal communication is inherently better than textual communication because the latter allows physicians time to think about the inquiry in great depth.

Communication comprises 2 primary processes according to the media synchronicity theory: the conveyance of information and the convergence of meaning [37]. Thus, we relate instrumental communication to the process of conveying information and associate affective communication with the process of achieving the convergence of meaning. The match between media capabilities and communication purposes could cause differences in communication performance. We further investigated the potential moderating effects of communication media (text vs voice) on the correlations between the communication features and economic returns of physicians. Therefore, we hypothesized the following: the associations between the instrumental communication features of physicians and their economic returns are moderated by the type of communication media (hypothesis 3), and the associations between the affective communication features of physicians and their economic returns are moderated by the type of communication media (hypothesis 4).

### Data Collection

**Figure 2:**
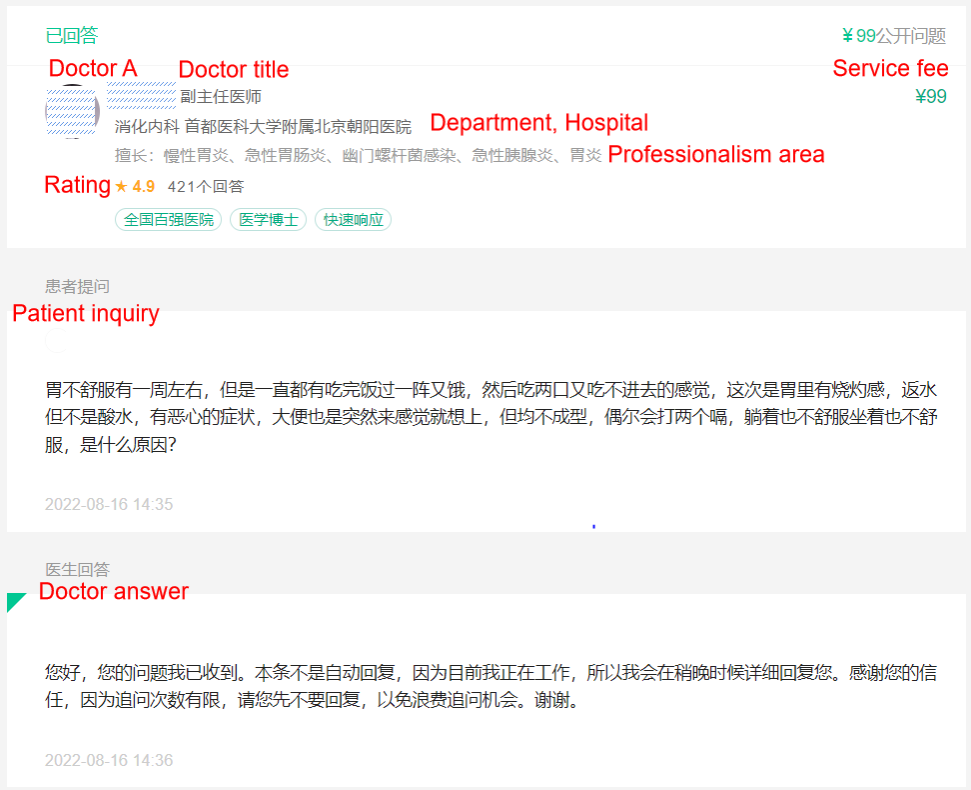
A sample consultation record page (accessed on August 29, 2022).

Objective secondhand data were collected from the Dingxiang Doctor website [4], a predominant web-based health consultation platform in China for >20 years. So far, the website serves >5.5 million active users and 2.1 million participating physicians. The Dingxiang Doctor website categorizes physicians into 39 disease specialties, which deliver different treatment solutions according to patients’ disease severity. We selected physicians who treat severe diseases (malignant tumors and heart disease) and who treat less severe diseases (digestive and endocrine diseases) to control interference from the disease type and severity [39]. We used a data crawling application to collect historical physician-patient consultation records. Each consultation instance contained several rounds of physician-patient interactions. Figure 2 presents a sample historical consultation record encompassing physician-related information (title, professional domain, disease department, hospital, and overall patient rating), patient consultation inquiries, physician’s replies, and consultation service charges. We collected data from 2 periods: June 2021 and December 2022. We excluded some duplicated or invalid samples containing errors and missing values and ultimately analyzed 40,563 consultation instances generated from 528 physicians between September 2016 and December 2022. Of the 528 physicians, 54 (10.2%) were directors, 154 (29.2%) were associate directors, 231 (43.8%) were attending physicians, and 89 (16.9%) were residents. The studied physicians had worked for an average of 14.34 (SD 7.01) years, and their average consultation fees were RMB 36.53 (US $5.10; SD 21.37).

### Ethical Considerations

Crawling techniques were used to collect data about web-based consultation from a public domain data set visible to all platform users. The studied consultations were not conducted for research purposes. The crawling program followed the Robots Exclusion Protocol. The home pages of the concerned physicians provided only the publicly available information of the physician (such as name, designations, and hospitals) and only the patient consultation information (content, time, and price) that patients had agreed to make public. The patient names were automatically anonymized on the home pages. In the data set handling process, we took precautionary measures to guarantee data security. We also applied to the institutional review board of Shenzhen University for the ethical review of the research project and obtained due approval (202300004) for the study protocol. The institutional review board had waived the requirement to obtain informed consent for this study.

### Dependent Variable

The payment received by individual physicians on a per-consultation basis was used to measure their economic returns. We used the log value of the returns in the empirical model. Notably, physicians could vary their consultation charges for different patients.

### Independent Variables

The instrumental and affective communication features were estimated from the messages transmitted by physicians for each consultation instance. We used a software named Textmind [31] to extract and quantify the instrumental and affective linguistic features as ratio values between 0 and 1. Textmind is a simplified Chinese-language analysis program developed based on LIWC and Chinese LIWC by the Computational Cyber-Psychology Lab at the Institute of Psychology at the Chinese Academy of Sciences [31]. It delivers a 1-stop solution from word segmentation to language feature analysis and has been effectively used in multiple Chinese-language studies [21,22].

### Moderating Variables

We classified the physician messages as textual and vocal communication by introducing an additional feature labeled as message media. Voice messages were assigned a value of 1, and textual messages were assigned a value of 0 [14].

### Control Variables

We controlled for the designations and working years of physicians, levels and rankings of their hospitals, development levels of cities in which the hospitals are located, and disease types. Physicians are accorded 4 designations on the Dingxiang Doctor website: director, associate director, attending physician, and resident physician. Physicians who are assigned high designations are assumed to have more experience in the treatment of particular diseases. Hospital types include private, public, and 3A. The term *3A* denotes tertiary hospitals equipped with more staff members and more patient beds (>501 beds) [40]. These hospitals are usually located in urban cities and provide high-level, specialized medical services. For example, a 3A hospital should set up at least 13 designated clinical departments and have >60 m^2^ of space on each floor [40]. Hospital rankings may also influence patient expectations. According to Fudan’s 2021 China hospital rankings [41], we classified institutions as ranked in the top 100 hospitals or not ranked in the top 100 hospitals. Disease types included malignant tumors, heart disease, digestive disease, and endocrine disease. The abovementioned control variables, apart from working years, were treated as categorical variables in the model estimating process. They are transformed into dummy variables.

Table 1 presents the descriptive statistics of all the variables, and the correlations among variables are reported in Multimedia Appendix 2. We used Spearman correlation instead of Pearson correlation because of the nonnormality of many LIWC indicators [25]. The correlations among variables (Multimedia Appendix 2) provide the basis for further analysis of the proposed model.

**Table 1:** Descriptive statistics.

Variable	Values, mean (SD; range)
Economic returns	36.527 (42.154; 1-699)
Insight	0.107 (0.088; 0-1.162)
Causation	0.058 (0.050; 0-0.643)
Discrepancy	0.144 (0.100; 0-1.551)
Tentativeness	0.121 (0.089; 0-1.083)
Certainty	0.033 (0.037; 0-0.754)
Positive emotion	0.078 (0.088; 0-1.048)
Anxiety	0.011 (0.021; 0-0.500)
Anger	0.002 (0.007; 0-0.200)
Sadness	0.004 (0.011; 0-0.387)
Voice service	0.090 (0.285; 0-1)
Working years	14.340 (7.908; 2-46)

## Results

### Model Estimation

We first conducted multiple linear regression analyses using SPSS (version 22; IBM Corp) to test our hypotheses about the impact of instrumental and affective communications on economic returns of physicians (log value). Table 2 reports the analysis results, which disclose that our independent variables and control variables explained 31.9% of the variance in the economic returns of physicians. The effect size (*f*^2^) is 0.468, and the regression model is significant at the 0.001 level. The variance inflation factor values ranged between 1.052 and 2.566, indicating that our study did not evince the problem of multicollinearity [42].

**Table 2:** Results of regression analysis^a,b^.

	B (SE)	β	*t* test (*df*)^c^	*P* value	VIF^d^
Constant	3.015 (0.006)	N/A^e^	516.813 (40,538)	<.001	N/A
Insight	0.248 (0.025)	.053	10.013 (40,538)	<.001	1.686
Causation	0.155 (0.039)	.019	4.012 (40,538)	<.001	1.326
Discrepancy	−0.294 (0.026)	−.072	−11.169 (40,538)	<.001	2.482
Tentativeness	0.189 (0.030)	.041	6.394 (40,538)	<.001	2.451
Certainty	0.528 (0.051)	.048	10.271 (40,538)	<.001	1.321
Positive emotion	0.283 (0.023)	.061	12.402 (40,538)	<.001	1.444
Anxiety	0.048 (0.083)	.002	0.574 (40,538)	.57	1.093
Anger	0.132 (0.253)	.002	0.520 (40,538)	.60	1.052
Sadness	−0.228 (0.153)	−.006	−1.489 (40,538)	.14	1.063
Working years	0.006 (0.000)	.118	17.930 (40,538)	<.001	2.566
Hospital rank	0.216 (0.004)	.239	52.578 (40,538)	<.001	1.235
Disease type	N/A	N/A	N/A	<.001	1.266
Physician rank	N/A	N/A	N/A	<.001	1.697
Hospital type	N/A	N/A	N/A	<.001	1.053
City tier	N/A	N/A	N/A	<.001	1.253

^a^*R*^2^=0.319.

^b^*F*_24_=792.686 (*P*<.001).

^c^2-tailed test.

^d^VIF: variance inflation factor.

^e^N/A: not applicable.

### Direct Effect

The results presented in Table 2 indicate that instrumental communication using words indicating insight (B=0.248; *P*<.001), causation (B=0.155; *P*<.001), tentativeness (B=0.189; *P*<.001), and certainty (B=0.528; *P*<.001) was positively associated with the economic returns of physicians. Affective communication using language expressing positive emotion (B=0.283; *P*<.001) was also linked with high economic returns. In contrast, the use of terms conveying discrepancy (B=−0.294; *P*<.001) by physicians was negatively related to their economic returns. Surprisingly, the use of words related to anxiety (B=0.048; *P*=.57), anger (B=0.132; *P*=.60), and sadness (B=−0.228; *P*=.14) did not affect the economic returns of physicians.

We also conducted 2 robustness tests to evaluate the results’ stability. First, because the feature values are small, we scaled the independent variables up by 100-fold and performed the multiple regression analysis again, which would not change the model’s fitness [43]. Second, we conducted quantile regression analysis to provide a more detailed analysis of the relationships between the communication features and economic returns of physicians. The results are reported in Multimedia Appendices 3 and 4, and they demonstrated the robustness of our multiple linear regression results.

### Moderating Effect

**Figure 3:**
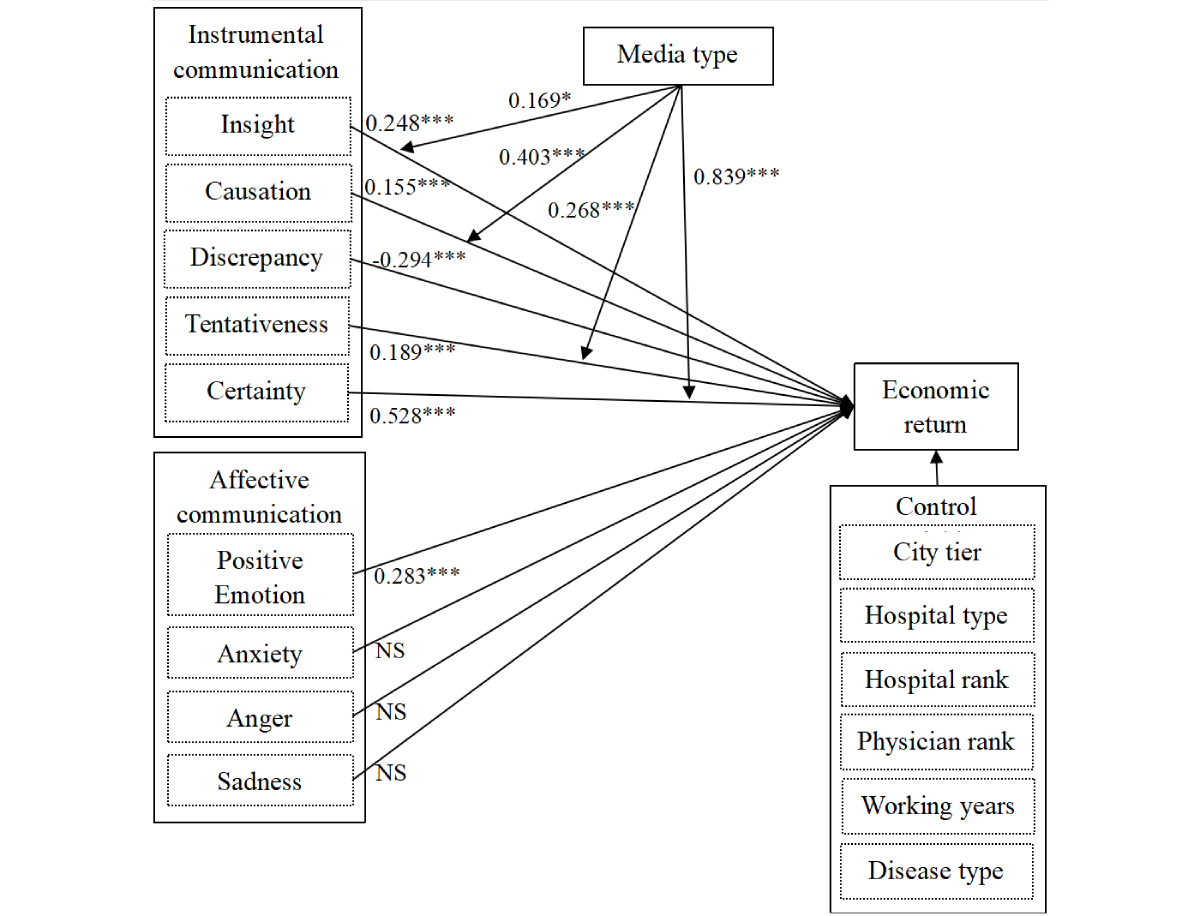
The results of main and moderating effects. The numbers indicate B values. NS: not significant; *P=.09; ***P<.001.

On the basis of the regression results, we further tested the moderating effects of communication media (text vs voice) using PROCESS Model 1 in SPSS (version 22) [44]. Table 3 displays the results of the moderating effects of communication media on significant influencing paths presented in Table 2. The findings reveal that communication media function significantly in moderating the influencing paths from the use of insight (B=0.169; *P*=.09), causation (B=0.403; *P*=.005), tentativeness (B=0.268; *P*<.001), and certainty (B=0.839; *P*<.001). However, communication media do not influence the use of words related to discrepancy (B=0.097; *P*=.21) and positive emotion (B=−0.159; *P*=.12). Figure 3 summarizes the results of the testing of our hypotheses.

**Table 3:** Summary of the moderating effects.

Test and path		B (SE)	*t* test (*df*)^a^	*P* value
**1**				
	Insight → economic returns	0.366 (0.030)	12.283 (40,551)	<.001
	Voice service → economic returns	0.044 (0.011)	3.925 (40,551)	<.001
	Insight×voice service → economic returns	0.169 (0.100)	1.680 (40,551)	.09
**2**				
	Causation → economic returns	0.310 (0.047)	6.575 (40,551)	<.001
	Voice service → economic returns	0.034 (0.011)	3.097 (40,551)	<.001
	Causation×voice service → economic returns	0.403 (0.145)	2.782 (40,551)	.005
**3**				
	Discrepancy → economic returns	−0.285 (0.032)	−9.026 (40,551)	<.001
	Voice service → economic returns	0.044 (0.013)	3.395 (40,551)	<.001
	Discrepancy×voice service → economic returns	0.097 (0.077)	1.262 (40,551)	.21
**4**				
	Tentativeness → economic returns	0.141 (0.036)	3.955 (40,551)	<.001
	Voice service → economic returns	0.024 (0.013)	1.858 (40,551)	.06
	Tentativeness×voice service → economic returns	0.268 (0.082)	3.285 (40,551)	<.001
**5**				
	Certainty → economic returns	0.586 (0.063)	9.311 (40,551)	<.001
	Voice service → economic returns	0.030 (0.010)	3.125 (40,551)	<.001
	Certainty×voice service → economic returns	0.839 (0.197)	4.260 (40,551)	<.001
**6**				
	Positive emotion → economic returns	0.242 (0.028)	8.777 (40,551)	<.001
	Voice service → economic returns	0.067 (0.009)	7.242 (40,551)	<.001
	Positive emotion×voice service → economic returns	−0.159 (0.102)	−1.558 (40,551)	.12

^a^2-tailed test.

**Figure 4:**
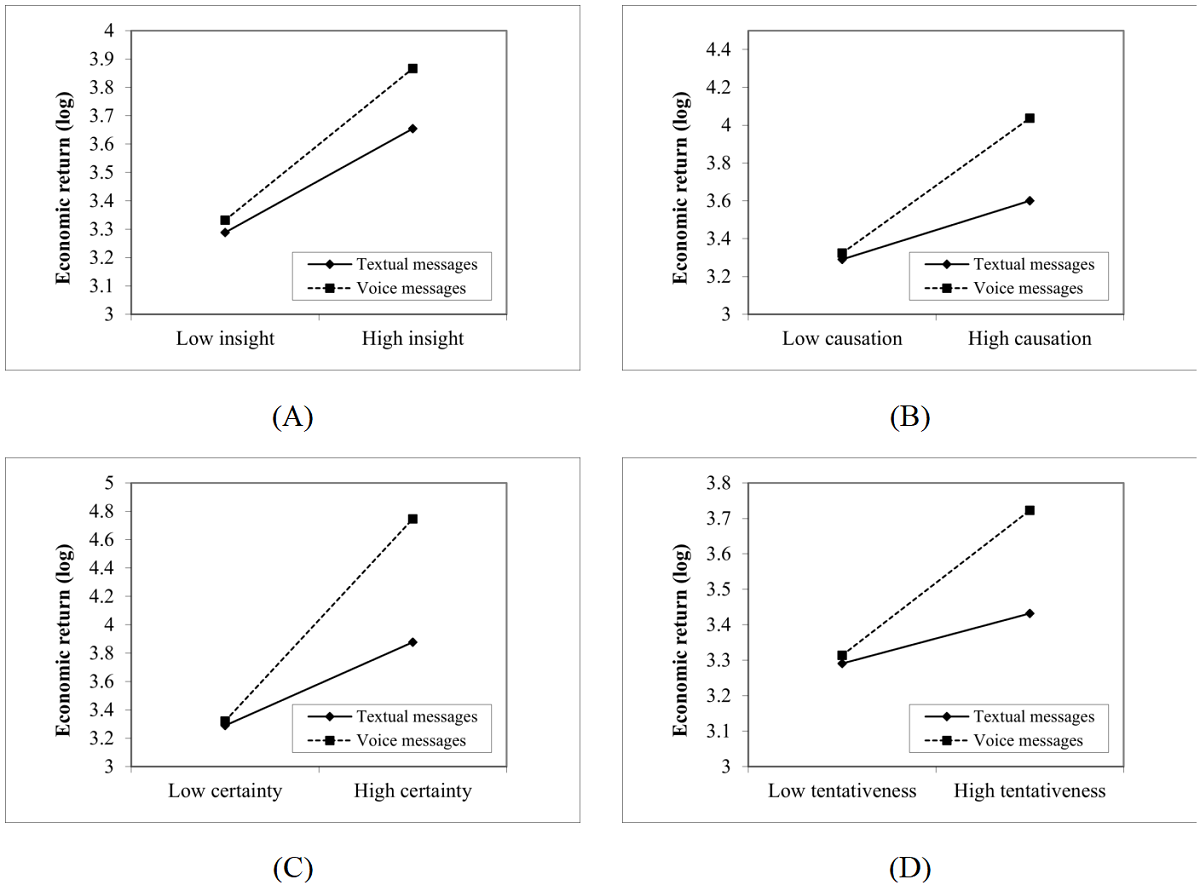
Results of the moderating effects.

Figure 4 further illustrates the moderating effects. We found that the use of voice messages could magnify the impact of insight-related words (simple slope=0.535; t_40,551_=5.324; *P*<.001), causation-related terms (simple slope=0.713; t_40,551_=5.055; *P*<.001), tentativeness-related terms (simple slope=0.409; t_40,551_=4.967; *P*<.001), and certainty-related terms (simple slope=1.425; t_40,551_=7.434; *P*<.001) on the economic returns of physicians. However, the use of textual and vocal messages did not vary the effects of terms communicating discrepancy and positive emotion.

### Communication Patterns

The regression results indicated that communication features exerted heterogeneous effects on the economic returns of physicians. In many cases, physicians offered both instrumental and affective responses to patients at different stages of the consultations. Thus, we investigated the compounding effects of discrete communicating features to attain deep insight into communication patterns. First, we transformed the physician responses into a sequence of messages according to the number of replies. The longest sequence comprised 16 messages. Second, we calculated the linguistic feature values for 9 psychological linguistic features for each message in the sequence and constructed a data set composed of 9×16 dimensions for every consultation instance. We entered 0 values for the remaining dimensions of consultations with sequence length <16, because no information was offered after the last message. Thus, the data set encompassed the 9 communication features at different stages of the consultations. Third, we used the K-means clustering method to cluster the consultation instances into multiple groups corresponding to discrete communication patterns. We then selected the optimal number of clusters by using ANOVA to compare the economic returns of physicians across the different groups. The T2 test by Tamhane [45] was selected for the analysis of the variance because the sample variance did not satisfy homogeneity. The results disclosed 4 clusters as the optimal number. Therefore, we obtained 4 groups of consultation instances associated with different levels of economic returns, as depicted in Multimedia Appendix 5.

Table 4 presents the detailed results of the T2 test by Tamhane [45]. The results elucidated that the third group commanded the highest economic returns, whereas the second group received the lowest gain. The mean revenue from consultations in the third group was 12.2% higher than the average earnings of the first group, 21.2% higher than the standard remunerations of the second group, and 14.6% higher than the average payments received by the fourth group.

**Table 4:** ANOVA results for economic returns (log)^a,b^.

Group	1			2			3			4	
	D (SE)	*P* value	D (SE)		*P* value	D (SE)		*P* value	D (SE)		*P* value
1	N/A^c^	N/A^b^	0.090 (0.026)		.004	–0.122 (0.010)		<.001	0.024 (0.009)		.03
2	–0.090 (0.026)	.004	N/A^b^		N/A^b^	–0.212 (0.026)		<.001	–0.066 (0.026)		.06
3	0.122 (0.010)	<.001	0.212 (0.026)		<.001	N/A^b^		N/A^b^	0.146 (0.009)		<.001
4	–0.024 (0.009)	.03	0.066 (0.026)		.06	–0.146 (0.009)		<.001	N/A^b^		N/A^b^

^a^The values in the table represent the difference (%) between the vertical and horizontal category labels.

^b^*F*_3_=87.971 (*P*<.001).

^c^N/A: not applicable.

To further elucidate the communication patterns of each group, we divided the 9 linguistic features into 3 dimensions according to the regression results: instrumental interactions, affective communication using positive emotions, and affective communication using negative emotions. As Multimedia Appendix 5 displays, we then visualized the communication features for each group using the mean values. The sequence length was set to 16 according to the longest sequence of physician messages. Multimedia Appendix 6 describes the visualization results for all 9 linguistic features.

**Figure 5:**
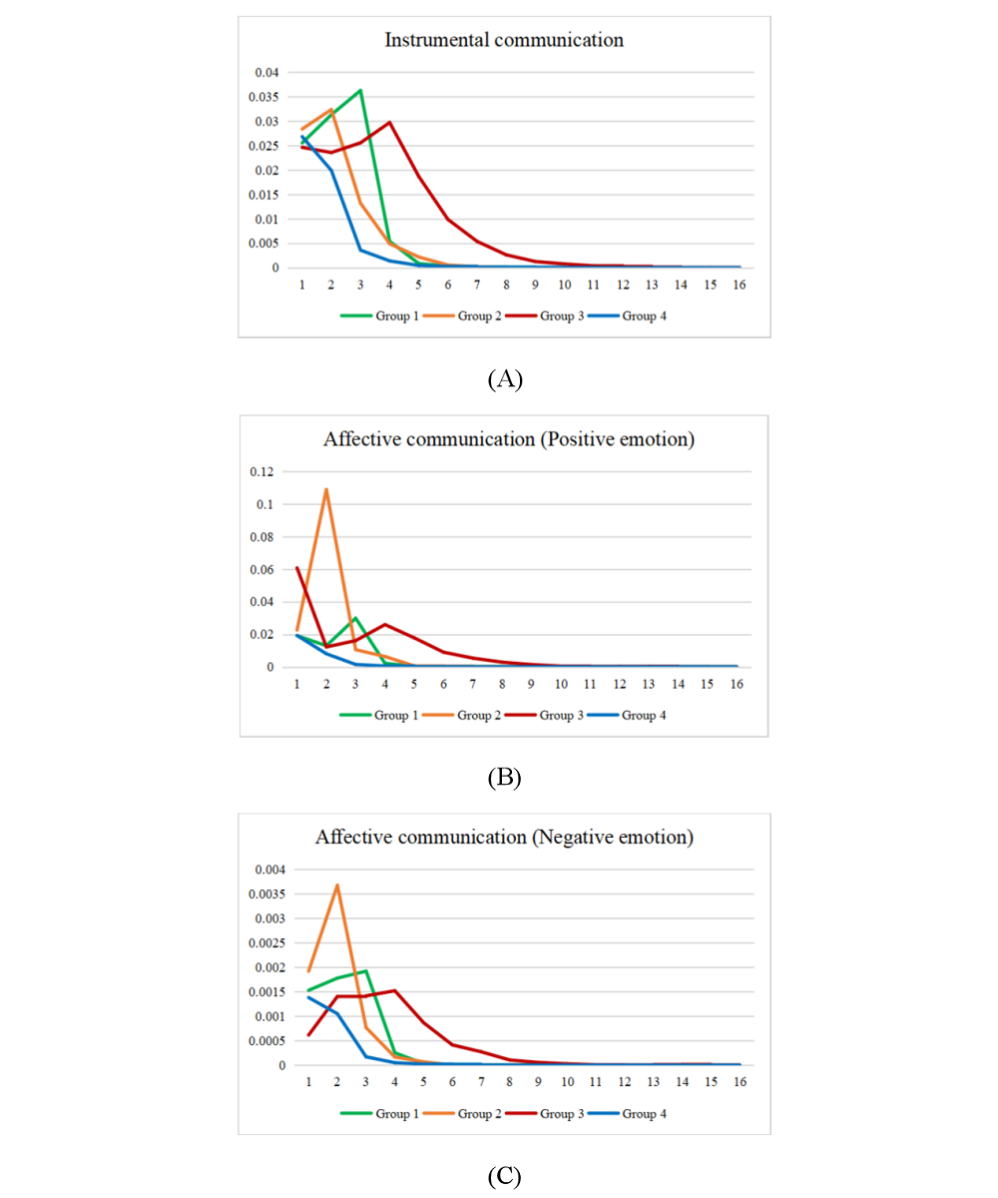
Distributions of communication features across the 4 groups: (A) instrumental communication, (B) affective communication (positive emotion), and (C) affective communication (negative emotion).

Figure 5 shows that consultations in the third group tended to include longer sequences than those categorized into other groups, signifying that physicians designated to the third group replied more frequently. These physicians also used more terms related to positive emotions at the beginning of their consultations and later offered more instrumental suggestions. In contrast, physicians in the second group used more terms related to positive and negative emotions, offered instrumental suggestions in their second reply, and then tended to end the consultation with a short response. Surprisingly, physicians in the fourth group used few emotion-related terms and provided moderate amounts of instrumental suggestions at the beginning of their consultations, and their economic returns were slightly higher than that of those in the second group.

## Discussion

### Principal Findings

This study aimed to investigate, at a more granular level, the associations between communication behaviors (instrumental and affective) and economic returns of physicians. We also explored the moderating effects of communication media (text vs voice). Our results indicate that the use of words indicating insight, causation, tentativeness, and certainty and the use of words indicating positive emotion were positively associated with the economic returns of physicians. In contrast, the use of terms conveying discrepancy by physicians was negatively related to their economic returns. The use of voice media by physicians intensified the impact of terms related to insight, causation, tentativeness, and certainty. The pattern analysis results indicate that physicians who responded to patients more frequently, communicated positive emotions at the beginning of the consultations, and provided more instrumental suggestions afterward achieved the highest economic returns.

Our findings align with the results reported in previous studies [2,6,7,14,46] and offer additional feature-level details. Moreover, the provision of logical and assured explanations helps reduce uncertainty in patients, who gain knowledge about the causes and consequences of their diseases. Specificity and clarity in expression and information delivery also improve communication quality [47]. Therefore, it is reasonable that physicians with superior instrumental communication skills achieve high economic returns. Moreover, patients may harbor great expectations from physicians with high consultation fees [6], which could drive physicians to devote more effort to cognitive thinking before providing answers. However, the increased use of discrepancy-related words (ie, should and would) could suggest that physicians notice divergences between their suggestions and patient behaviors and highlight them during conversations. Thus, they may unintentionally deliver dissatisfaction signals to patients. Hence, instrumental communication using discrepancy-related words was negatively associated with the economic returns of physicians.

Our results also indicate that affective communication encompassing more terms related to positive emotions (ie, happy, love, and nice) was positively linked with high economic returns of physicians. This result is congruent with previous findings that the enhancement of service quality mandates the delivery of emotional support for patients [8,11,33], which is also a vital patient-centered communication skill [48]. The patients’ psychological needs for empathy and care are satisfied when physicians demonstrate compassion and encouragement [7]. Consequently, physicians with better affective communication skills tend to receive high economic returns. However, the articulation of negative emotions such as anger, anxiety, and sadness was not related to the economic returns attained by physicians. Perhaps, the use of words conveying anger, anxiety, and sadness by physicians could deliver negative signals to patients and undermine their confidence in their physicians, even though such terms could also transmit empathy. Physicians are suggested to use language related to understanding, respecting, and supporting to express empathy to patients [47].

The moderating effects analysis revealed that the choice of text or voice media for communication can moderate the influence exerted by certain linguistic features. The media synchronicity theory posits that the use of media supporting high synchronicity and multiplicity of cues is more suited to complex communication [37]. Explaining the causes of diseases or syndromes during web-based consultations may be deemed as information transmission processes with high requirements for media synchronicity. Therefore, such messages become more significantly influential through voice media, which is characterized by high synchronicity [38]. These findings suggest that physicians who make smart use of voice media to communicate with patients tend to obtain high economic returns.

Our pattern analysis results unveiled the compounding effects of multiple communication features. Providing positive emotional support to patients at the beginning of the consultation can fulfill the psychological needs of patients before satisfying their knowledge requirements [47]. This process enhances the outcomes of instrumental communication because patients may find it difficult to comprehend disease knowledge and treatment suggestions if they are emotionally occupied. Therefore, consultations that first delivered positive emotional support and later supplied the instrumental comments were found to yield high levels of economic returns. This partially aligns with the previous finding that interaction frequency positively affects patient satisfaction [19]. In contrast, consultations that simultaneously offered emotional support and instrumental comments in a limited number of replies yielded low levels of economic returns. These findings imply that physicians who address the emotional needs of patients before offering professional advice are more likely to obtain high economic returns.

### Limitations and Future Directions

Despite the contributions of our study, we must indicate a few limitations. First, our data were collected from the Dingxiang Doctor website in China. The generalization of our study’s findings in other countries would require us to obtain data from multiple websites in many other countries. Second, although our data contain a relatively large set of consultation cases, we should collect data sets encompassing long time durations and generate a panel data set that would guarantee the robustness of our findings. Third, we did not include patients’ personal preferences in our conceptual model such as patients’ education level because of the limited access to patients’ personal information. We believe future study that investigates the impact of patients’ preferences will contribute novel insight into this research issue. Finally, we can expand our study by relating the communication features of physicians to their medical domain knowledge to provide deep insight into the service quality of web-based health care consultations.

### Conclusions

This study demonstrates that the economic returns of physicians are associated with their communication features and the media used for web-based health care consultations. This study adopted a psychological and linguistic perspective to offer methodological referential value for relevant prospective studies of web-based physician-patient interactions. Moreover, it supplements the limited literature relating to the economic returns received by physicians through web-based health care platforms. The findings deliver important practical directions for improving the quality of web-based consultation services provided by physicians.

## Appendix

Multimedia Appendix 1 Linguistic features related to cognitive and affective processes.

Multimedia Appendix 2 Pair-wise correlations (Spearman correlation).

Multimedia Appendix 3 Regression results (independent variables×100).

Multimedia Appendix 4 Results of quantile regression analysis.

Multimedia Appendix 5 Levels of economic returns across the 4 groups.

Multimedia Appendix 6 Distribution of communication features.

## Abbreviations

LIWC: Linguistic Inquiry and Word Count
