# Supplementary material for: The Association Between Linguistic Characteristics of Physicians’ Communication and Their Economic Returns: Mixed Method Study
Source: J Med Internet Res. 2024 Jan 11;26:e42850. doi: 10.2196/42850 (PMC10811595; doi:10.2196/42850)
Supplement: Multimedia Appendix 1 [file jmir_v26i1e42850_app1.docx]

Appendix 1. Linguistic features related to cognitive and affective processes

| **SC-LIWC categories** | **Features** | **Example words** |
| --- | --- | --- |
|  |  |  |
| Cognition process | Insight | Think, know |
|  | Causation | Because, effect |
|  | Discrepancy | Should, would |
|  | Tentative | Maybe, perhaps |
|  | Certainty | Always, never |
| Affective process | Positive emotion | Happy, love, nice, sweet |
|  | Anxiety | Worried, fearful |
|  | Anger | Hate, kill, annoyed |
|  | Sad | Crying, grief, sad |
