# Supplementary material for: The Association Between Linguistic Characteristics of Physicians’ Communication and Their Economic Returns: Mixed Method Study
Source: J Med Internet Res. 2024 Jan 11;26:e42850. doi: 10.2196/42850 (PMC10811595; doi:10.2196/42850)
Supplement: Multimedia Appendix 2 [file jmir_v26i1e42850_app2.docx]

Appendix 2. Pair-wise correlations (Spearman)^[[1]](#footnote-0)^

|  |  | Economic Return | Insight | Causation | Discrepancy | Tentative | Certainty | Positive Emotion | Anxiety | Anger | Sad |
| --- | --- | --- | --- | --- | --- | --- | --- | --- | --- | --- | --- |
| Economic Return | Coefficient | 1 |  |  |  |  |  |  |  |  |  |
|  | P value |  |  |  |  |  |  |  |  |  |  |
| Insight | Coefficient | 0.133^***^ | 1 |  |  |  |  |  |  |  |  |
|  | P value | <.001 |  |  |  |  |  |  |  |  |  |
| Causation | Coefficient | 0.111^***^ | 0.402^***^ | 1 |  |  |  |  |  |  |  |
|  | P value | <.001 | <.001 |  |  |  |  |  |  |  |  |
| Discrepancy | Coefficient | 0.054^***^ | 0.395^***^ | 0.389^***^ | 1 |  |  |  |  |  |  |
|  | P value | <.001 | <.001 | <.001 |  |  |  |  |  |  |  |
| Tentative | Coefficient | 0.080^***^ | 0.421^***^ | 0.360^***^ | 0.739^***^ | 1 |  |  |  |  |  |
|  | P value | <.001 | <.001 | <.001 | <.001 |  |  |  |  |  |  |
| Certainty | Coefficient | 0.145^***^ | 0.397^***^ | 0.303^***^ | 0.339^***^ | 0.329^***^ | 1 |  |  |  |  |
|  | P value | <.001 | <.001 | <.001 | <.001 | <.001 |  |  |  |  |  |
| PosEmo | Coefficient | 0.116^***^ | 0.513^***^ | 0.243^***^ | 0.327^***^ | 0.306^***^ | 0.387^***^ | 1 |  |  |  |
|  | P value | <.001 | <.001 | <.001 | <.001 | <.001 | <.001 |  |  |  |  |
| Anxiety | Coefficient | 0.082^***^ | 0.219^***^ | 0.175^***^ | 0.195^***^ | 0.169^***^ | 0.175^***^ | 0.184^***^ | 1 |  |  |
|  | P value | <.001 | <.001 | <.001 | <.001 | <.001 | <.001 | <.001 |  |  |  |
| Anger | Coefficient | 0.081^***^ | 0.132^***^ | 0.142^***^ | 0.108^***^ | 0.125^***^ | 0.132^***^ | 0.125^***^ | 0.115^***^ | 1 |  |
|  | P value | <.001 | <.001 | <.001 | <.001 | <.001 | <.001 | <.001 | <.001 |  |  |
| Sad | Coefficient | 0.063^***^ | 0.144^***^ | 0.134^***^ | 0.110^***^ | 0.139^***^ | 0.138^***^ | 0.111^***^ | 0.125^***^ | 0.315^***^ | 1 |
|  | P value | <.001 | <.001 | <.001 | <.001 | <.001 | <.001 | <.001 | <.001 | <.001 |  |

1. ** P < .1; ** P< .05; *** P< .01; n.s. = not significant* [↑](#footnote-ref-0)
