# Supplementary material for: The Association Between Linguistic Characteristics of Physicians’ Communication and Their Economic Returns: Mixed Method Study
Source: J Med Internet Res. 2024 Jan 11;26:e42850. doi: 10.2196/42850 (PMC10811595; doi:10.2196/42850)
Supplement: Multimedia Appendix 3 [file jmir_v26i1e42850_app3.docx]

Appendix 3. Regression results (Independent variables *100)^[[1]](#footnote-0)^

|  | ***B*** | ***SE*** | ***β*** | ***t*** | ***P*** | **VIF** |
| --- | --- | --- | --- | --- | --- | --- |
| Constant | 3.015 | 0.006 | / | 516.813 | <.001 | / |
| Insight | 0.002 | 0.000 | 0.053 | 10.013 | **<.001** | 1.686 |
| Causation | 0.002 | 0.000 | 0.019 | 4.012 | **<.001** | 1.326 |
| Discrepancy | -0.003 | 0.000 | -0.072 | -11.169 | **<.001** | 2.482 |
| Tentative | 0.002 | 0.000 | 0.041 | 6.394 | **<.001** | 2.451 |
| Certainty | 0.005 | 0.001 | 0.048 | 10.271 | **<.001** | 1.321 |
| Positive emotion | 0.003 | 0.000 | 0.061 | 12.402 | **<.001** | 1.444 |
| Anxiety | 0.000 | 0.001 | 0.002 | 0.574 | 0.566 | 1.093 |
| Anger | 0.001 | 0.003 | 0.002 | 0.520 | 0.603 | 1.052 |
| Sad | -0.002 | 0.002 | -0.006 | -1.489 | 0.137 | 1.063 |
| Working years | 0.006 | 0.000 | 0.118 | 17.930 | <.001 | 2.566 |
| Hospital rank 100 | 0.216 | 0.004 | 0.239 | 52.578 | <.001 | 1.235 |
| Disease type | / | / | / | / | <.001 | 1.266 |
| Doctor rank | / | / | / | / | <.001 | 1.697 |
| Hospital type | / | / | / | / | <.001 | 1.053 |
| City tier | / | / | / | / | <.001 | 1.353 |
| R^2^ | 0.319 | | | | | |
| F | 792.686*** | | | | | |

1. ** P < .1; ** P< .05; *** P< .01; n.s. = not significant* [↑](#footnote-ref-0)
