# Supplementary material for: The Association Between Linguistic Characteristics of Physicians’ Communication and Their Economic Returns: Mixed Method Study
Source: J Med Internet Res. 2024 Jan 11;26:e42850. doi: 10.2196/42850 (PMC10811595; doi:10.2196/42850)
Supplement: Multimedia Appendix 4 [file jmir_v26i1e42850_app4.docx]

Appendix 4. Results of quantile regression analysis^[[1]](#footnote-0)^

|  | Quantile 0.25 | Quantile 0.5 | Quantile 0.75 |
| --- | --- | --- | --- |
| Constant | 1.604*** | 2.270*** | 2.401*** |
|  | (0.042) | (0.095) | (0.071) |
| Insight | 0.066*** | 0.426*** | 0.398*** |
|  | (0.013) | (0.030) | (0.022) |
| Causation | 0.109*** | 0.117** | -0.014 |
|  | (0.021) | (0.047) | (0.035) |
| Discrepancy | -0.076*** | -0.298*** | -0.370*** |
|  | (0.014) | (0.032) | (0.024) |
| Tentative | 0.081*** | 0.204*** | 0.174*** |
|  | (0.016) | (0.036) | (0.027) |
| Certainty | 0.155*** | 0.457*** | 0.585*** |
|  | (0.028) | (0.062) | (0.046) |
| Positive emotion | 0.076*** | 0.326*** | 0.312*** |
|  | (0.012) | (0.028) | (0.021) |
| Anxiety | 0.056 | -0.151 | 0.148** |
|  | (0.045) | (0.101) | (0.075) |
| Anger | -0.069 | 0.158 | 0.145 |
|  | (0.136) | (0.307) | (0.278) |
| Sad | -0.056 | -0.374 | -0.199 |
|  | (0.082) | (0.186) | (0.138) |
| Working years | 0.000 | 0.006*** | 0.007*** |
|  | (0.000) | (0.000) | (0.000) |
| Hospital rank 100 | 0.236*** | 0.276*** | 0.188*** |
|  | (0.002) | (0.005) | (0.004) |
| Disease type | /*** | /*** | /*** |
| Doctor rank | /*** | /*** | /*** |
| Hospital type | /*** | /*** | /*** |
| City tier | /*** | /*** | /*** |
| Pseudo R^2^ | 0.087 | 0.197 | 0.245 |
| MAE | 0.344 | 0.268 | 0.321 |

1. ** P < .1; ** P< .05; *** P< .01; n.s. = not significant* [↑](#footnote-ref-0)
