# Supplementary figures and images for: The Association Between Linguistic Characteristics of Physicians’ Communication and Their Economic Returns: Mixed Method Study
Source: J Med Internet Res. 2024 Jan 11;26:e42850. doi: 10.2196/42850 (PMC10811595; doi:10.2196/42850)

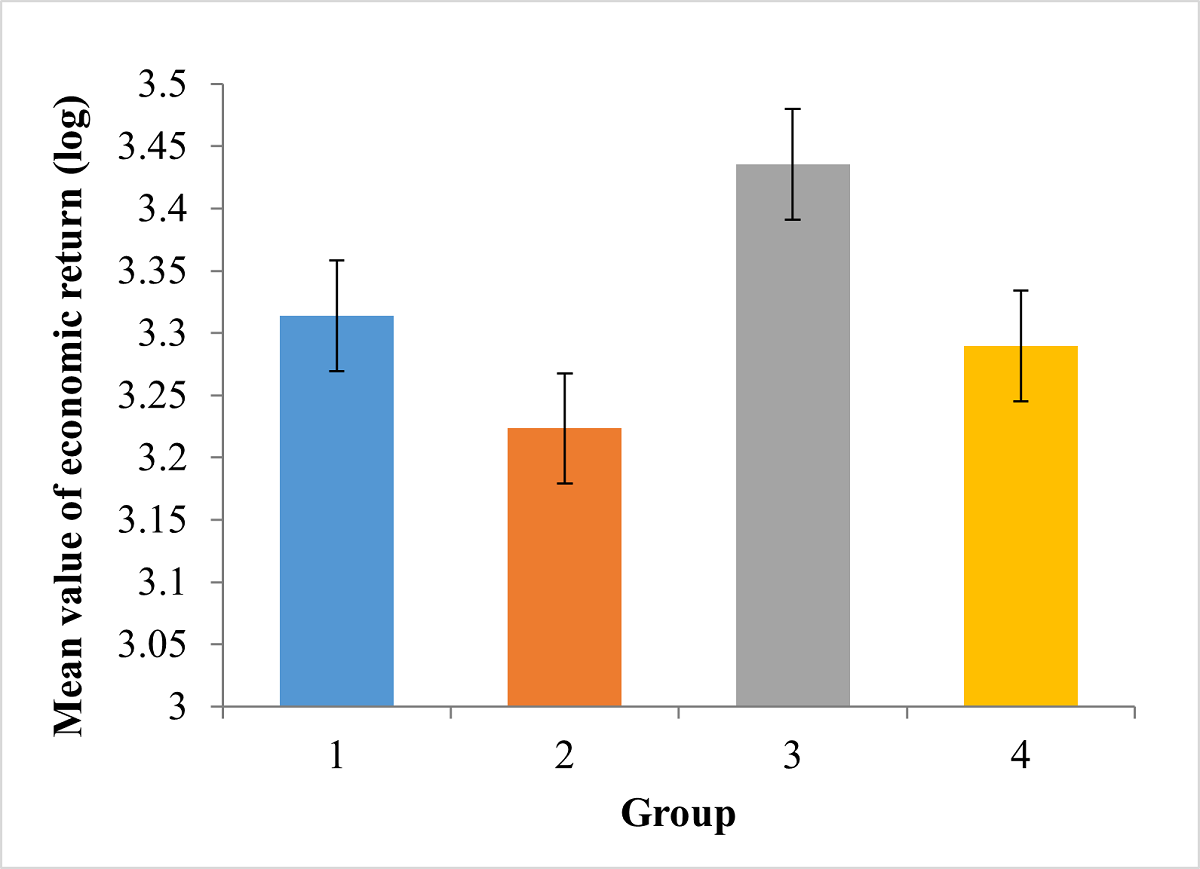

Supplement: Multimedia Appendix 5 [file jmir_v26i1e42850_app5.png]
