# Supplementary material for: The Association Between Linguistic Characteristics of Physicians’ Communication and Their Economic Returns: Mixed Method Study
Source: J Med Internet Res. 2024 Jan 11;26:e42850. doi: 10.2196/42850 (PMC10811595; doi:10.2196/42850)
Supplement: Multimedia Appendix 6 [file jmir_v26i1e42850_app6.docx]

Appendix 5. Distribution of communication features

|  |  |  |
| --- | --- | --- |
| (a) | (b) | (c) |
|  |  |  |
| (d) | (e) | (f) |
|  |  |  |
| (g) | (h) | (i) |
